# Diagnosis-Related Group Payment Systems and Hospital Efficiency, Quality, and Coding Behavior: A Systematic Review and Narrative Synthesis

**DOI:** 10.7759/cureus.116701

**Published:** 2026-09-22

**Authors:** Ayman Mahgoub, Mahdi Aljaber, Mohamed Ibrahim Mustafa, Ahmed Alazrak, Wael Alsebaei

**Affiliations:** 1 Revenue Cycle Management, Saudi German Hospital, Makkah, SAU; 2 Revenue Cycle Management, Saudi German Hospital, Jeddah, SAU; 3 Medical Operation, Bupa Arabia, Jeddah, SAU; 4 Clinical Governance, Bupa Arabia, Jeddah, SAU; 5 Revenue Cycle Management, Al Salama Hospital, Jeddah, SAU

**Keywords:** ar-drg, coding behavior, diagnosis-related groups, hospital efficiency, hospital quality, length of stay, prospective payment system, saudi billing system, upcoding, value-based healthcare

## Abstract

Diagnosis-related group (DRG)-based payment systems are used globally to incentivize hospital efficiency, yet their impact on clinical quality and documentation behavior remains variable. As Saudi Arabia implements the Australian Refined DRGs (AR-DRG) framework under Vision 2030, a synthesis of international evidence is required to inform sustainable rollout. This review examined the effects of DRG-based payment on hospital efficiency, quality, and coding behavior in studies published between 2010 and 2025. We searched PubMed, Scopus, Web of Science, Google Scholar, and the Saudi Digital Library for English-language reports published between January 1, 2010, and July 31, 2025. Only primary comparative or quasi-experimental studies were eligible for the evidence synthesis; systematic reviews and meta-analyses were used only for contextual comparison. Two reviewers independently performed screening, risk-of-bias assessment (Risk Of Bias In Non-randomized Studies of Interventions (ROBINS-I) for difference-in-differences and other quasi-experimental designs; Cochrane Effective Practice and Organisation of Care criteria for interrupted time series), and data extraction; findings were synthesized narratively because of heterogeneity. Six primary comparative studies from Germany, the United States, England, and China met the eligibility criteria. DRG-based payment was associated with a shorter average length of stay (LOS) in most but not all settings, with reductions reported in England and China, whereas the German national reform showed no systematic country-level reduction. Cost per case generally fell in regional pilot studies, with significant reductions observed in specific urban settings. Quality outcomes were mixed: 30-day readmission rates remained stable or decreased in specific pilot implementations, while mortality signals were inconsistent across settings. Strategic coding behavior was evident in some mature systems, where upcoding accounted for a substantial portion of the growth in high-intensity discharge categories, whereas coding-related incentives were associated with reduced provision of low-value services in other settings. Using Grading of Recommendations Assessment, Development, and Evaluations (GRADE), certainty was Moderate for the reduction in LOS and Very Low for readmission; other outcomes were not formally assessed. DRG-based payment is associated with reductions in average LOS in many settings, but its effects on clinical quality are heterogeneous and context-dependent. Robust quality monitoring and coding-audit frameworks should be considered and prospectively evaluated alongside any DRG implementation. For Saudi Arabia, prospective evaluation of the AR-DRG framework and the Saudi Billing System rollout, using pre-specified efficiency and quality indicators, is needed before conclusions can be drawn about local outcomes.

## Introduction and background

The global transition toward prospective, case-based payment systems has redefined hospital financing over the past four decades. The diagnosis-related group (DRG) framework, which categorizes patients into clinically coherent groups with similar resource consumption, stands at the center of this shift [1]. By shifting financial risk to providers through fixed-rate reimbursement, DRGs incentivize cost containment and process standardization [2]. Since its inception at Yale University, the model has diffused from United States Medicare to Europe and, increasingly, to emerging economies seeking value-based healthcare reform [3]. 

The central economic rationale for DRGs is the moderation of hospital cost inflation by discouraging the volume-driven incentives of fee-for-service models [2]. However, the international literature highlights a complex balance between efficiency and quality. While reductions in length of stay (LOS) are frequently reported, concerns persist regarding “quicker and sicker” discharges and the potential for upcoding, in which cases are strategically assigned to higher-paying categories to maximize revenue [4,5]. These unintended consequences depend heavily on the implementation context, digital maturity, and the presence of rigorous auditing [6]. 

In the Kingdom of Saudi Arabia (KSA), the health system is undergoing fundamental restructuring under the Vision 2030 Health Sector Transformation Program [7]. The Ministry of Health is transitioning to a strategic-purchaser role, organizing healthcare into semi-autonomous clusters [8]. To support this model, KSA has adopted the Australian Refined DRGs (AR-DRG) framework and launched the Saudi Billing System (SBS). The methodological development and validation of the SBS code set have been described in a national implementation study [9]. Integrated through the National Platform for Health and Insurance Exchange Services (NPHIES), these reforms aim to enhance billing specificity and transparency [10]. 

This review is distinctive in bringing together comparative DRG evidence on efficiency, quality, and coding behavior and in explicitly translating that evidence to the Saudi reform context, where AR-DRG and the SBS are being implemented at national scale. Given the scale of the Saudi rollout, a systematic appraisal of the international experience is essential. This review evaluates the global impact of DRG-based payment between 2010 and 2025 across four domains: operational efficiency, clinical quality, financial and behavioral responses (upcoding), and practical implications for Saudi Arabian health reform.

## Review

Protocol, registration, and reporting 

This systematic review was conducted and reported in accordance with the Preferred Reporting Items for Systematic Reviews and Meta-Analyses (PRISMA) 2020 statement [11]. A review protocol defining the research question, eligibility criteria, and extraction template was prepared a priori. The review question was framed using the Population, Intervention, Comparator, Outcome, and Study design (PICOS) structure: among acute-care hospitals or hospitalized patients (Population), how does the implementation or modification of a DRG-based payment system (Intervention), compared with fee-for-service, per diem, or a pre-implementation baseline (Comparator), affect hospital efficiency, financial performance, clinical quality, and coding behavior (Outcomes), as evaluated in comparative or quasi-experimental studies (Study design)? 

The protocol was prepared a priori but was not registered in PROSPERO. No substantive deviations from the planned methods occurred. 

Eligibility criteria 

Studies were eligible if they: (1) evaluated the impact of a DRG-based payment system or one of its major variants (for example, AR-DRG, All Patient Refined DRGs, G-DRG, or Medicare Severity DRGs) on hospital performance; (2) used a comparative or quasi-experimental design, including difference-in-differences, interrupted time series, controlled before-after, propensity-score matching, or cohort analysis, in which DRG payment was compared with a non-DRG or pre-DRG control condition; (3) reported at least one quantitative outcome of efficiency (e.g., LOS or cost per case), quality (e.g., readmission or mortality), or coding behavior (e.g., DRG distribution or coded severity); and (4) were published in English between January 1, 2010, and July 31, 2025.

These sources describe the Saudi reform infrastructure and are cited only as contextual and background material; they were not counted among the formally included studies. Systematic reviews and meta-analyses of DRG impact were likewise not included in the formal synthesis; instead, they were retained as contextual sources and are cited in the Discussion for comparison of pooled estimates. Case reports, editorials, conference abstracts without extractable data, outpatient-only or primary-care payment studies, and reports based on overlapping datasets already captured by an included study were excluded. Contextual policy reports, methodological development and code-validation studies (for example, the SBS development study by Reka et al. 2025 [9]), government documents, and gray literature did not meet the comparative-design criterion and were used only for narrative interpretation in the Introduction and Discussion. 

Information sources and search strategy 

Five sources were searched from January 1, 2010, to July 31, 2025: PubMed, Scopus, Web of Science, Google Scholar (the first 200 records, sorted by relevance), and the Saudi Digital Library. The search combined controlled vocabulary and free-text terms for the intervention and outcomes. For illustration, the PubMed strategy was: ("Diagnosis-Related Groups"[Mesh] OR "diagnosis related group*"[tiab] OR DRG[tiab] OR "case-based payment*"[tiab] OR "prospective payment system*"[tiab]) AND ("Length of Stay"[Mesh] OR "length of stay"[tiab] OR cost*[tiab] OR efficiency[tiab] OR readmission*[tiab] OR mortality[tiab] OR upcoding[tiab] OR "coding creep"[tiab]). Analogous strategies were constructed and adapted for Scopus (TITLE-ABS-KEY syntax), Web of Science (Topic [TS] field tags), Google Scholar, and the Saudi Digital Library; the full, reproducible search strategy for every database, including platform interface, field tags, and filters, is reported in Appendix A. The reference lists of included reviews were also hand-searched. 

The systematic search was last conducted on July 31, 2025, and no studies were added to the evidence base after that date. Three website references [7,8,10] carry access dates of July 10, 2026, reflecting URL verification during manuscript preparation; their content was unchanged since the search closure on July 31, 2025. The Google Scholar search was limited to the first 200 records, sorted by relevance, applied consistently across all search iterations. 

Study selection 

All records were imported into a reference manager and de-duplicated. Two reviewers, AM (Ayman Mahgoub) and AA (Ahmed Alazrak), independently screened titles and abstracts against the eligibility criteria, independently assessed the full texts of potentially eligible records, and extracted data using a piloted extraction form. Disagreements at either stage were resolved through discussion and, when consensus could not be reached, by consultation with a third reviewer, MJA (Mahdi Jamal Aljaber). Reasons for full-text exclusion were recorded and are reported in the study selection flow (Figure 1). 

**Figure 1:**
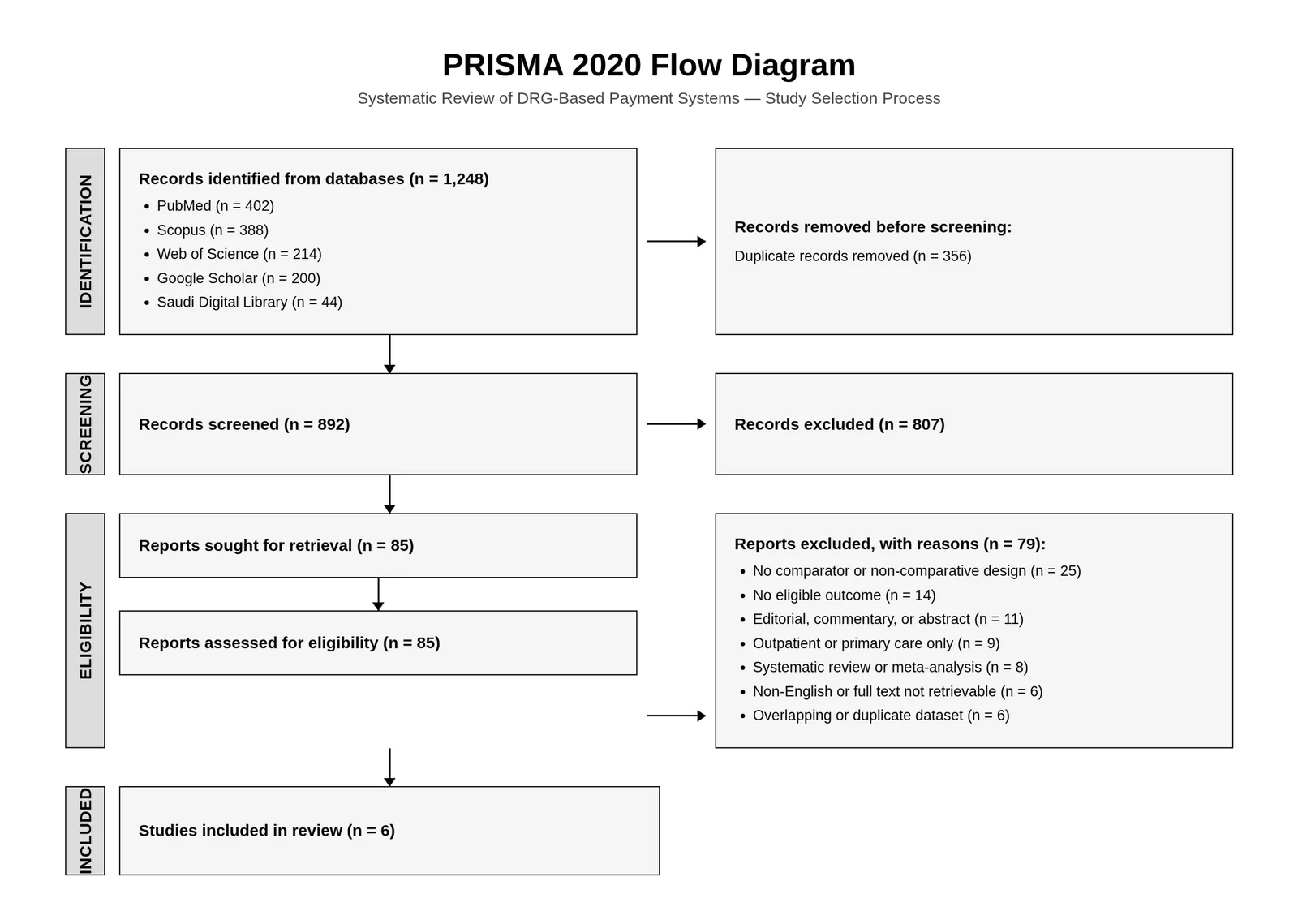
PRISMA 2020 flow diagram of study selection DRG, diagnosis-related group; PRISMA, Preferred Reporting Items for Systematic Reviews and Meta-Analyses.

Data extraction 

Data were extracted independently by two reviewers using a piloted extraction form and were cross-checked for accuracy. Extracted items included citation details, country and setting, study period, sample size or scope, DRG model, comparator, study design, outcome definitions, effect estimates with CIs where available, and reported limitations. Where a study was described in more than one report, the linked reports were treated as one study. 

Risk-of-bias assessment 

Two reviewers independently assessed risk of bias, with disagreements resolved by consensus. For difference-in-differences and other quasi-experimental (non-randomized) studies of interventions, we used the ROBINS-I tool, which appraises seven bias domains -- confounding, selection of participants into the study, classification of interventions, deviations from intended interventions, missing data, measurement of outcomes, and selection of the reported result -- with each domain rated as low, moderate, serious, or critical risk of bias (or no information) and an overall judgment derived across domains [12]. For interrupted time series studies, we applied the Cochrane Effective Practice and Organisation of Care (EPOC) suggested risk-of-bias criteria, which assess whether the intervention was independent of other changes, whether the shape of the intervention effect was pre-specified, whether the intervention was unlikely to affect data collection, blinding of outcome assessment, completeness of outcome data, protection against selective outcome reporting, and other risks of bias [13]. Studies were not excluded solely on the basis of higher risk of bias; instead, findings were interpreted cautiously, and lower-risk studies were given greater interpretive weight. Domain-level risk-of-bias ratings for all included studies are reported in Appendix B.

Data synthesis 

Because the included studies differed substantially in DRG model, payment context, study design, and outcome definitions, a meta-analysis of the primary studies was not undertaken. Findings were synthesized narratively following the Synthesis Without Meta-analysis (SWiM) reporting guideline [14], grouped by outcome domain: efficiency, quality, coding behavior, and implementation context. Effect measures were pre-specified a priori in the protocol: LOS as a percentage change or standardized mean difference (SMD); cost as a percentage change or mean difference in the reported currency and price-year; readmission and mortality as risk ratios (RRs), odds ratios, or percentage-point changes; and coding behavior as the change in the distribution of coded severity or the proportion of high-intensity discharges. The certainty of evidence for the two most homogeneous outcomes (LOS and readmission) was assessed using the Grading of Recommendations, Assessment, Development, and Evaluations (GRADE) framework. The potential for reporting and publication bias was formally considered during synthesis: because the review was restricted to English-language publications and formal small-study tests were not feasible given the narrative approach, reporting bias could not be excluded and is treated as a source of downgrading in the GRADE assessment and is revisited among the limitations. Formal assessment of reporting bias (e.g., funnel plot asymmetry) was not feasible given the small number of included studies and the heterogeneity of outcome measures. 

Search results and study selection 

The systematic search identified 1,248 records across five databases (PubMed: 402; Scopus: 388; Web of Science: 214; Google Scholar: 200; Saudi Digital Library: 44). After 356 duplicates were removed, 892 records were screened by title and abstract, and 807 were excluded as irrelevant. The full texts of 85 reports were assessed for eligibility, and 79 were excluded, with reasons detailed in Figure 1 (no comparator or non-comparative design, n=25; no eligible outcome, n=14; editorial, commentary, or abstract, n=11; outpatient or primary care only, n=9; systematic review or meta-analysis, n=8; non-English or full text not retrievable, n=6; overlapping or duplicate dataset, n=6). Six primary comparative or quasi-experimental studies met all eligibility criteria and were included in the synthesis. Systematic reviews, meta-analyses, policy briefs, government reports, methodological development studies, and industry white papers that were consulted during the search were used exclusively as contextual or background material and were not entered into the synthesis of effects. The selection process is summarized in the PRISMA 2020 flow diagram (Figure 1). 

Characteristics of included evidence 

The included evidence base comprises six primary comparative or quasi-experimental studies from high-income and middle-income health systems in Germany, the United States, England, and China. The distribution of the included studies by region, study design, and outcome/cost domain is summarized in Figure 2. The designs comprised difference-in-differences analyses (Messerle and Schreyögg [6]; Aragón et al. [15]; Cao et al. [16]), an observational regression analysis (Crespin et al. [4]), and interrupted time series analyses (Li et al. [17]; Zou et al. [18]). The characteristics of the included studies are presented in Table 1 (with expanded details in Appendix C), and a summary of principal quantitative findings by domain is presented in Table 2.

**Figure 2:**
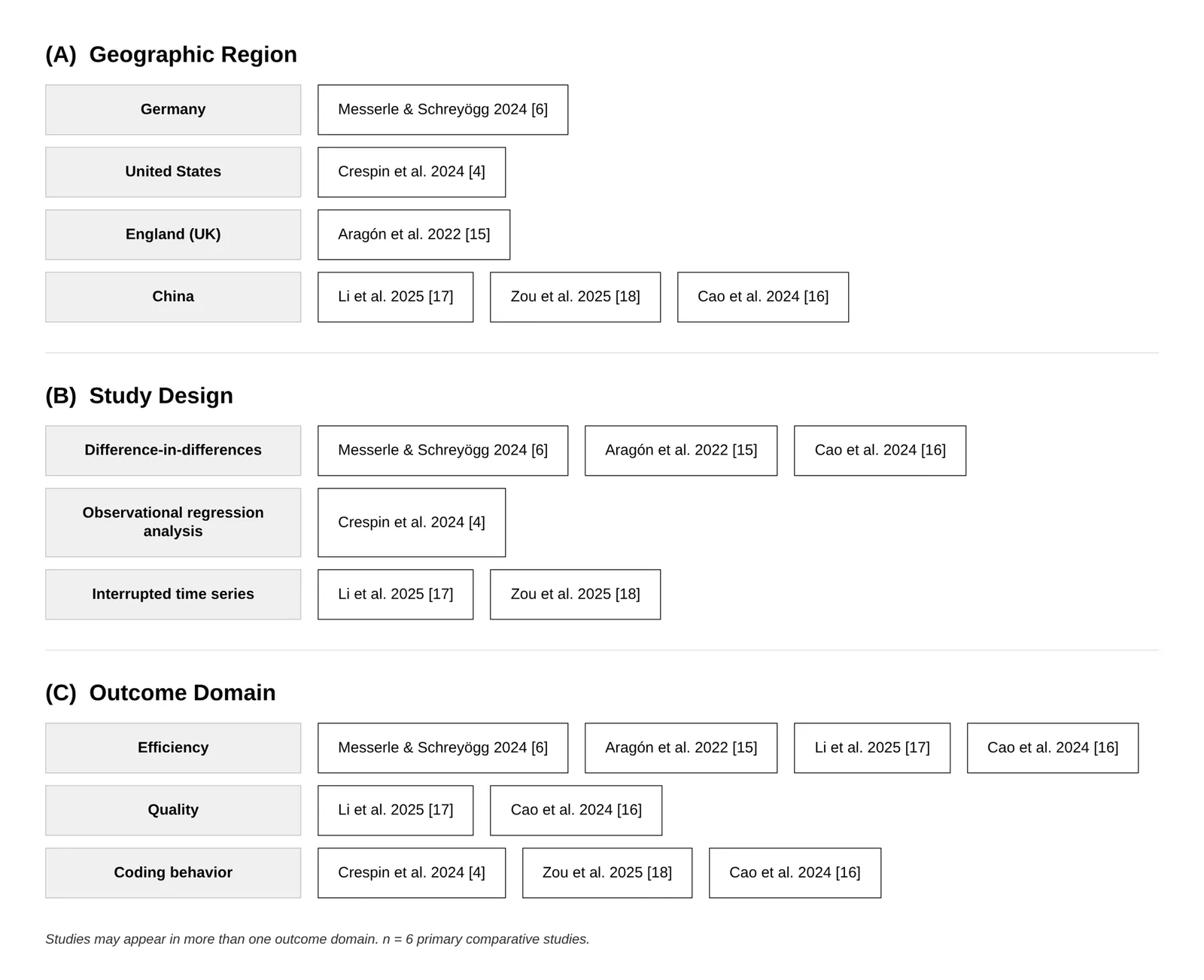
Evidence map of included studies (n=6) by (A) geographic region, (B) study design, and (C) outcome domain

**Table 1:** Characteristics of the six included primary studies Note: Cost and price-year data are reported where available in the original sources. C-DRG: Chinese diagnosis-related group; CI: confidence interval; DRG: diagnosis-related group; EPOC: Cochrane Effective Practice and Organisation of Care; G-DRG: German DRG; HRG: Healthcare Resource Group; ITS: interrupted time series; LOS: length of stay; MS-DRG: Medicare Severity DRG; NHS: National Health Service; OECD: Organisation for Economic Co-operation and Development; PbR: Payment by Results; ROBINS-I: Risk Of Bias In Non-randomized Studies of Interventions.

#	First author, year	Country/region	Study design	Study period	DRG system	Sample/scope	Outcome domain	Key findings	Quality rating
1	Messerle and Schreyögg, 2024 [6]	Germany	Difference-in-differences (country-level)	~1 decade (national data)	G-DRG	National OECD data	Efficiency/system reform	Hospital activity rose ~20% over a decade; LOS did not decrease systematically at the country level.	Moderate (ROBINS-I)
2	Crespin et al., 2024 [4]	United States (five states)	Observational regression analysis	2011-2019	MS-DRG	~37.9 million discharges, 2011-2019	Coding behavior/upcoding	Upcoding explained up to two-thirds of the growth in highest-intensity discharges; ~US$14.6 billion in additional payments in 2019	Moderate (ROBINS-I)
3	Aragón et al., 2022 [15]	England (UK)	Difference-in-differences (Scotland control)	~15 years (NHS data)	HRG (Payment by Results)	15 years of NHS administrative micro-data	Efficiency	LOS fell significantly, and the effect grew over time; day-case admissions increased	Low (ROBINS-I)
4	Li et al., 2025 [17]	China (Meishan; 29 pilot hospitals)	Interrupted time series	2017-2022	Chinese DRG payment reform	1,617,608 insurance records, 2017-2022	Efficiency; quality	LOS: 0.56%/month, total costs -1.23%/month, cost-sharing -1.46%/month (2019-2020); 30-day readmission not significantly changed (-0.11%/month; 95% CI: -0.73% to 0.50%)	Low risk (EPOC)
5	Zou et al., 2025 [18]	China	Interrupted time series	January 2017 to December 2020	C-DRG	Coronary heart disease patients	Coding behavior/service appropriateness	Immediate reduction in low-value coronary revascularization of -9.78% (95% CI: -11.08% to -8.48%); sustained quarterly decrease of -0.59% (95% CI: -0.88% to -0.30%)	Low risk (EPOC)
6	Cao et al., 2024 [16]	China (Beijing; 53 hospitals)	Difference-in-differences	March 2021 to October 2022	Chinese DRG-based prepayment	Patients with neurologic disorders	Efficiency; quality; coding behavior	Total costs per case: -12.6%; LOS: -0.96 days; in-hospital mortality and readmissions reduced; a lower share of surgical cases and a lower case mix index suggest possible patient selection.	Moderate (ROBINS-I)

**Table 2:** Summary of principal quantitative findings by outcome domain Estimates are drawn from the six included primary studies and should be interpreted cautiously because of heterogeneity across systems, years, and outcome definitions. Pooled estimates from systematic reviews are discussed separately as contextual comparisons. C-DRG: Chinese diagnosis-related group; CI: confidence interval; GRADE: Grading of Recommendations Assessment, Development, and Evaluations; LOS: length of stay.

Outcome domain	Reported estimate	Source	Interpretive caveat
Length of stay	Reductions in England (grew over time) and China (Meishan LOS: -0.56%/month; Beijing: -0.96 days per case); no systematic country-level reduction in Germany	[6,15-17]	Effect present in most but not all settings. GRADE certainty: Moderate
Cost per case	Total hospital costs: -1.23%/month (Meishan); total costs per case: -12.6% (Beijing)	[16,17]	Individual admission costs generally constrained; system-wide costs may rise with volume.
Readmission	No significant change in Meishan (-0.11%/month; 95% CI: -0.73% to 0.50%); reduction reported in Beijing	[16,17]	Direction of effect inconsistent across settings. GRADE certainty: Very low
Mortality	Inconsistent; reduction in the Beijing study, no consistent signal elsewhere	[16]	Certainty not formally assessed (very low confidence)
Coding behavior	Upcoding explained up to two-thirds of growth in highest-intensity US discharges (~US$14.6 billion in 2019); low-value coronary revascularization fell under C-DRG in China (-9.78% immediate; -0.59%/quarter)	[4,18]	Setting-specific; certainty not formally assessed (very low confidence)

Impact on operational efficiency 

The reduction of average LOS was the most frequently reported efficiency outcome across the primary studies. In England, Aragón et al. [15] found that the long-run introduction of Healthcare Resource Group-based Payment by Results was associated with a significant and persistent reduction in LOS that grew over time. In China, Li et al. [17], in a large interrupted time series across 29 pilot hospitals in Meishan, found that LOS decreased by 0.56% per month during the first reform period (2019-2020), alongside monthly reductions in total hospital costs (1.23%) and patient cost-sharing (1.46%), with effects stabilizing in 2021-2022; a Beijing quasi-experimental study of patients with neurologic disorders (Cao et al. [16]) reported that LOS fell by 0.96 days and total costs per case decreased by 12.6%. However, these effects were not universally observed: the German national reform (Messerle and Schreyögg [6]) indicated that, while hospital activity increased by approximately 20% over a decade, LOS did not decrease systematically at the country level. Taken together, DRG-based payment tended to constrain individual admission costs and LOS in most but not all settings, although system-wide expenditures may still rise if admission volumes increase. 

Impact on clinical quality

Evidence regarding clinical quality outcomes, such as readmission and mortality, was mixed and varied by setting. In the Meishan interrupted time series (Li et al. [17]), 30-day readmission did not change significantly (-0.11% per month; 95% CI: -0.73% to 0.50%). Cao et al. [16] reported significant reductions in both in-hospital mortality and readmission rates, although a concurrent fall in the proportion of surgical cases and a decline in the case mix index raised the possibility of patient selection. Mortality findings were otherwise inconsistent across the evidence base, with no consistent signal in either direction. These divergent results indicate that the direction and magnitude of quality effects depend heavily on the DRG variant, the baseline payment system, and the local implementation context. 

Financial behavior and coding documentation (DRG creep) 

Behavioral responses to DRG incentives were evident in the primary studies. Crespin et al. [4], analyzing approximately 37.9 million discharges across five US states, attributed up to two-thirds of the growth in the highest-intensity discharges to changes in coding behavior rather than patient acuity, corresponding to an estimated US$14.6 billion in additional payments in 2019. This estimate is setting-specific and should not be generalized across countries or payment systems. In contrast, Zou et al. [18], in a Chinese interrupted time series examining the C-DRG system, found an immediate reduction in low-value coronary revascularization of 9.78% (95% CI: -11.08% to -8.48%) and a sustained quarterly decrease of 0.59% (95% CI: -0.88% to -0.30%), indicating that DRG incentives can also curb the provision of low-value services. The terms upcoding and DRG creep are related but not identical: upcoding refers to the intentional assignment of cases to higher-paying categories to maximize revenue, whereas DRG creep denotes a broader, potentially unintentional or systemic drift toward higher-coded severity that may also reflect improved documentation practices; the two are distinguished here to avoid conflation. 

Certainty of evidence (GRADE) 

Using the GRADE framework, the certainty of evidence began at low for all outcomes, reflecting the observational, non-randomized nature of the evidence base. For LOS, certainty was rated Moderate. Starting from low, the rating was upgraded for a large and consistent effect observed across diverse health systems (England and China) and for a plausible dose-response relationship, whereby greater financial pressure was associated with shorter stays. It was not upgraded to high because the German national reform (Messerle and Schreyögg [6]) showed no systematic country-level reduction in LOS, indicating that the effect is not uniform across all settings. For readmission, certainty was rated Very Low. Starting from low, the rating was downgraded for serious inconsistency, because the direction of effect was contradictory across studies (no significant change in the Meishan interrupted time series (Li et al. [17]) versus a reduction in the Beijing study (Cao et al. [16])), and for serious indirectness, because the effect depended heavily on the DRG variant, the baseline payment system, and the local context; imprecision was an additional concern given the wide CIs in individual studies. For the remaining outcomes, including mortality and coding behavior, certainty was not formally assessed and should be regarded as reflecting very low confidence. Formal GRADE assessment was restricted to the two outcomes -- LOS and 30-day readmission -- for which the evidence base permitted defensible outcome-level judgments across multiple studies. For the remaining outcomes (cost and financial performance, coding behavior, and implementation context), the diversity of study designs, outcome definitions, and measurement approaches precluded formal certainty rating; these outcomes were assessed as very low confidence based on the overall evidence profile. A summary of the certainty of evidence for these principal outcomes is provided in Table 3. 

**Table 3:** GRADE summary of findings for the principal outcomes of DRG-based payment Certainty of evidence was formally assessed with GRADE for length of stay and readmission only; ratings for cost per case, mortality, and coding behavior are not formal GRADE assessments and are reported as very low confidence. CI: confidence interval; C-DRG: Chinese diagnosis-related group; GRADE: Grading of Recommendations, Assessment, Development, and Evaluations; LOS: length of stay.

Outcome	Number of studies	Certainty of evidence (GRADE)	Summary of findings
LOS	4 [6,15-17]	Moderate	Consistent reductions in England and China (Meishan LOS: -0.56%/month; Beijing: -0.96 days per case); no systematic country-level reduction in Germany
Readmission (30 days)	2 [16,17]	Very low	Mixed and inconclusive: no significant change in the Meishan interrupted time series (-0.11%/month; 95% CI: -0.73% to 0.50%); reduction reported in the Beijing study
Cost per case	2 [16,17]	Very low (not formally assessed)	Reductions in per-case cost in China (Meishan total costs: -1.23%/month; Beijing: -12.6%); system-wide costs may rise if admission volumes increase.
Mortality	1 [16]	Very low (not formally assessed)	Inconsistent; a reduction was reported in the Beijing study, with no consistent signal elsewhere.
Coding behavior	2 [4,18]	Very low (not formally assessed)	Setting-specific: upcoding explained up to two-thirds of growth in highest-intensity US discharges (~US$14.6 billion in 2019), whereas low-value coronary revascularization fell under the Chinese C-DRG system.

Implications for the Saudi Arabian context 

Methodological development work on the SBS, cited here as contextual/background material rather than as included comparative evidence [9], reported a 30% expansion of the code set (1,774 new codes; 7,947 codes in total) to address specificity gaps, with 25% of sampled records requiring new or alternative codes during validation. The digital-first architecture of NPHIES may, in principle, support more complete and auditable documentation, but this has not yet been evaluated against efficiency or quality outcomes in the Saudi setting; the international evidence highlights a critical need for clinical coding certification and digital infrastructure maturity across all health clusters [8,9]. 

This systematic review synthesized evidence published between 2010 and 2025 on the impact of DRG-based payment systems, drawing on six primary comparative studies. The findings suggest that, while DRG implementation is associated with improvements in hospital operational efficiency -- primarily through reduced LOS -- the effects on clinical quality are heterogeneous and highly dependent on the implementation context [6,16,17]. These patterns are broadly consistent with the pooled estimates reported in earlier systematic reviews and meta-analyses, which are discussed below as contextual comparisons [5,19-21]. The identified trade-offs, such as potential pressure on readmission and the prevalence of upcoding in mature systems, highlight the necessity of robust monitoring and audit frameworks [4]. 

Interpretation and mechanism 

The efficiency gains observed are consistent with the theoretical incentives of prospective payment, which rewards hospitals for cost-effective care within fixed case rates. The magnitude of the length-of-stay reductions observed in the English and Chinese primary studies is broadly in line with pooled estimates from earlier systematic reviews, which reported an average LOS reduction of 8.07% (95% CI: 3.10-13.05) under DRG-based payment compared with cost-based systems [5] and a lower LOS corresponding to a SMD of -0.25 in a second review [19]. A further review of case-based payment in China similarly reported reduced inpatient expenditure and LOS [21]. However, the German experience (Messerle and Schreyögg [6]) is a reminder that the magnitude of any gain depends on the baseline payment model and the specific design of the reform. For readmission, the contextual reviews are themselves divergent -- one meta-analysis reported a small increase (1.36%; 95% CI: 0.45-2.27) [5] while another found no significant change (RR: 0.79; 95% CI: 0.62-1.01) [19], and a broad review of activity-based funding pointed toward increased discharges to post-acute care (RR: 1.24; 95% CI: 1.18-1.31) [20] -- mirroring the mixed signals in the primary evidence and supporting the hypothesis that efficiency pressures can, in some settings, lead to premature discharges that require careful quality oversight. A strength of this review is the restriction of its formal synthesis to primary comparative studies, together with duplicate screening and duplicate data extraction; the synthesis also connects international DRG evidence to an active national reform setting, improving practical relevance. 

Implications for Saudi Arabia’s Vision 2030 

For the Saudi Health Sector Transformation Program, the adoption of AR-DRG and the SBS provides an infrastructure for value-based purchasing [3,8]. The added specificity of the SBS and its integration with NPHIES may help narrow the coding ambiguities that can facilitate upcoding, although whether this translates into measurable gains has not yet been evaluated in the Saudi context [9,10]. The international evidence suggests that sustained investment in coding capacity and the development of quality-linked payment qualifiers (for example, readmission penalties) will be essential to sustain the integrity of the new system. Whether the Kingdom realizes the efficiency and quality benefits observed in some international settings will depend on factors that require direct, prospective evaluation. Implications should be read in descending order of confidence: DRGs often shorten LOS (higher confidence); quality outcomes are mixed and require monitoring (moderate confidence); coding behavior can respond strategically to financial incentives (higher confidence); and long-term Saudi outcomes remain uncertain and require direct evaluation (lower confidence). These implications should be interpreted as informed extrapolation from international evidence; direct evaluation of the AR-DRG and SBS rollout in the Saudi context is needed before local conclusions can be drawn. 

Future research 

Priority questions for future primary research include the following. What is the effect of AR-DRG implementation on 30-day readmissions in Saudi hospitals? Does SBS coding granularity reduce upcoding or shift it to other documentation behaviors? Which cluster-level governance features best protect quality while preserving efficiency gains? And how does DRG adoption affect rural-urban equity in access and outcomes? 

Limitations of the evidence and review process 

This review has several limitations that should be considered when interpreting the findings. First, because of substantial clinical and methodological heterogeneity among the six included primary studies, results were synthesized narratively rather than through a de novo meta-analysis; pooled estimates from previous systematic reviews are cited only as contextual comparisons [5,19]. Second, formal GRADE certainty ratings were generated only for the two most homogeneous outcomes (LOS, rated Moderate, and readmission, rated Very Low); for the remaining outcomes, the heterogeneity of study designs and outcome definitions did not permit defensible outcome-specific certainty judgments, and certainty for those outcomes should be regarded as reflecting very low confidence. 

Third, a chronological point warrants clarification: the systematic search was conducted and closed on July 31, 2025, whereas three website-based references [7,8,10] carry access dates of July 10, 2026, reflecting final URL verification during manuscript preparation rather than the search date; the substantive content of these references was unchanged between the two dates, and no new evidence was added to the synthesis after the search closure. Fourth, the review was limited to English-language publications, and reporting and publication bias could not be formally excluded; non-English DRG literature (particularly from Germany, France, and Southeast Asia) may not be fully captured. Fifth, the Google Scholar search was pragmatic, limited to the first 200 results, which may have missed lower-ranked relevant studies. The small number of included studies (n=6) limits the precision of outcome-specific conclusions and precluded formal meta-analysis. The evidence base comprised only six primary studies from four countries (Germany, the United States, England, and China), with no representation from low- or middle-income countries outside China or from the Middle East, including Saudi Arabia. This geographic concentration limits the generalizability of the findings, and the policy implications for the Kingdom should be interpreted as informed extrapolation from international evidence rather than direct evidence of local effects. Finally, the review protocol was not registered in PROSPERO. Although the protocol was prepared a priori, the absence of prospective registration means that protocol adherence cannot be independently verified. Retrospective registration was not pursued because the review had already progressed beyond the screening stage when registration was considered. 

## Conclusions

DRG-based payment is associated with reductions in average LOS in many settings, but its success is context-dependent and its effects are not universal. Implementation is frequently accompanied by heterogeneous quality outcomes, including mixed signals on readmission, and a risk of strategic coding behavior that can erode financial gains in mature systems. For Saudi Arabia, the combination of AR-DRG and the SBS offers a structured framework for health-system reform under Vision 2030. To help ensure success, policymakers should pair the transition with robust clinical auditing, quality-linked incentives, and ongoing investment in digital maturity and coding expertise, and should evaluate the rollout prospectively with pre-specified efficiency and quality indicators. These lessons may also guide other health systems transitioning toward prospective, case-based reimbursement. 

## Appendices

Appendix A

Full Database Search Strategies 

All five databases were searched from January 1, 2010, to July 31, 2025 (search last run July 31, 2025). Each strategy was adapted from the PubMed master strategy shown below, preserving the two conceptual blocks (intervention terms AND outcome terms). Filters were limited to English language and the 2010-2025 publication window where the interface allowed. 

PubMed (MEDLINE): searched via pubmed.ncbi.nlm.nih.gov 

("Diagnosis-Related Groups"[Mesh] OR "diagnosis related group*"[tiab] OR DRG[tiab] OR "case-based payment*"[tiab] OR "prospective payment system*"[tiab]) AND ("Length of Stay"[Mesh] OR "length of stay"[tiab] OR cost*[tiab] OR efficiency[tiab] OR readmission*[tiab] OR mortality[tiab] OR upcoding[tiab] OR "coding creep"[tiab]). Filters: English; 2010/01/01-2025/07/31. 

Scopus: searched via scopus.com (advanced search, TITLE-ABS-KEY) 

( TITLE-ABS-KEY ( "diagnosis related group*" OR "DRG" OR "case-based payment*" OR "prospective payment system*" ) AND TITLE-ABS-KEY ( "length of stay" OR cost* OR efficiency OR readmission* OR mortality OR upcoding OR "coding creep" ) ) AND ( LIMIT-TO ( LANGUAGE , "English" ) ) AND PUBYEAR > 2009 AND PUBYEAR < 2026. 

Web of Science Core Collection: searched via webofscience.com (Topic [TS] field) 

TS=("diagnosis related group*" OR "DRG" OR "case-based payment*" OR "prospective payment system*") AND TS=("length of stay" OR cost* OR efficiency OR readmission* OR mortality OR upcoding OR "coding creep"). Refined by: Languages = English; Publication Years = 2010-2025. 

Google Scholar: searched via scholar.google.com 

("diagnosis related group" OR DRG OR "case-based payment" OR "prospective payment system") AND ("length of stay" OR cost OR efficiency OR readmission OR mortality OR upcoding OR "coding creep"). Custom range 2010-2025; results screened to the first 200 records sorted by relevance; patents and citations excluded. 

Saudi Digital Library (SDL): searched via sdl.edu.sa (unified/EDS interface) 

AB ("diagnosis related group*" OR "DRG" OR "case-based payment*" OR "prospective payment system*") AND AB ("length of stay" OR cost* OR efficiency OR readmission* OR mortality OR upcoding OR "coding creep"). Limiters: English language; peer-reviewed; date published 2010-2025. 

Appendix B

Domain-Level Risk-of-Bias Assessments 

Domain-level risk-of-bias assessments for the six included primary studies. Difference-in-differences and other quasi-experimental (non-randomized) studies were appraised with the ROBINS-I tool (Panel A), which evaluates seven bias domains together with an overall judgment (Low, Moderate, Serious, or Critical risk of bias). Interrupted time series studies were appraised with the Cochrane EPOC suggested risk-of-bias criteria for interrupted time series designs (Panel B), covering seven criteria that are each rated as low risk, high risk, or unclear. These ratings are consistent with the quality assessment column of Table 1 in the main manuscript. 

**Table 4:** Quasi-experimental studies (difference-in-differences and regression): ROBINS-I risk-of-bias domains

ROBINS-I domain	Messerle and Schreyögg, 2024 [6]	Crespin et al., 2024 [4]	Aragón et al., 2022 [15]	Cao et al., 2024 [16]
Confounding	Moderate	Moderate	Low	Moderate
Selection of participants into the study	Low	Low	Low	Moderate
Classification of interventions	Low	Low	Low	Low
Deviations from intended interventions	Low	Low	Low	Low
Missing data	Low	Low	Low	Low
Measurement of outcomes	Low	Low	Low	Low
Selection of the reported result	Low	Low	Low	Low
Overall risk of bias	Moderate	Moderate	Low	Moderate

**Table 5:** Interrupted time series studies: Cochrane EPOC risk-of-bias criteria

Cochrane EPOC risk-of-bias criterion (interrupted time series)	Li et al., 2025 [17]	Zou et al., 2025 [18]
Intervention independent of other changes	Low risk	Low risk
Shape of the intervention effect pre-specified	Low risk	Low risk
Intervention unlikely to affect data collection	Low risk	Low risk
Knowledge of the intervention adequately prevented (blinding of outcome assessment)	Low risk (objective outcomes; blinding not applicable)	Low risk (objective outcomes; blinding not applicable)
Incomplete outcome data adequately addressed	Low risk	Low risk
Free from selective outcome reporting	Low risk	Low risk
Free from other risk of bias	Low risk	Low risk
Overall risk of bias	Low risk	Low risk

ROBINS-I domains are rated as Low, Moderate, Serious, or Critical risk of bias, or No information, with an overall risk-of-bias judgment. Cochrane EPOC interrupted time series criteria are each rated as low risk, high risk, or unclear. Ratings correspond to the quality assessment column of Table 1 in the main manuscript. Risk-of-bias tool references: Sterne JAC, Hernan MA, Reeves BC, et al. ROBINS-I: a tool for assessing risk of bias in non-randomized studies of interventions. BMJ. 2016;355:i4919 [12]. Cochrane Effective Practice and Organisation of Care: Suggested risk of bias criteria for EPOC reviews. (2017) [13]. 

Appendix C

*Characteristics of Eligible Included Studies* 

Comparative and quasi-experimental primary studies that met the review eligibility criteria (English-language studies published January 1, 2010 - July 31, 2025 evaluating a DRG-based payment system on hospital efficiency, quality, or coding behavior). Six primary comparative or quasi-experimental studies were included. 

**Table 6:** Characteristics of eligible included studies C-DRG, Chinese diagnosis-related group; CI, confidence interval; DID, difference-in-differences; DRG, diagnosis-related group; EPOC, (Cochrane) Effective Practice and Organisation of Care; G-DRG, German diagnosis-related group; HRG, Healthcare Resource Group; ITS, interrupted time series; LOS, length of stay; MS-DRG, Medicare severity diagnosis-related groups; NHS, National Health Service; OECD, Organisation for Economic Co-operation and Development; PbR, Payment by Results; ROBINS-I, Risk Of Bias In Non-randomized Studies of Interventions; USD, United States dollar.

Study	Country/setting	Design	DRG system	Sample/population	Key outcomes	Quality assessment tool	Overall rating
Messerle and Schreyögg, 2024 [6]	Germany; national hospital system	Difference-in-differences (quasi-experimental)	G-DRG	National OECD and German hospital-system data	Hospital activity increased following the reform; length of stay did not decrease systematically at the national level. Outcome domains: efficiency.	ROBINS-I	Moderate
Crespin et al., 2024 [4]	United States (5 states)	Observational regression analysis	MS-DRG	~37.9 million hospital discharges	Upcoding explained up to two-thirds of the growth in the highest-intensity discharges; additional DRG-based payments were estimated at $14.6 billion in 2019 (nominal USD). The estimate is setting-specific. Outcome domains: coding behavior.	ROBINS-I	Moderate
Aragón et al., 2022 [15]	England, United Kingdom (Scotland as control)	Difference-in-differences (Scotland control)	HRG under Payment by Results (PbR)	15 years of NHS administrative hospital micro-data	HRG-based Payment by Results was associated with a significant and persistent reduction in length of stay that grew over time, alongside an increased proportion of day-case admissions. Outcome domains: efficiency.	ROBINS-I	Low
Li et al., 2025 [17]	Meishan, China (29 pilot hospitals), 2017-2022	Interrupted time series (ITS)	Chinese DRG payment reform	1,617,608 medical insurance records	First reform period (2019-2020): total hospital costs decreased 1.23%/month, patient cost-sharing decreased 1.46%/month, and length of stay decreased 0.56%/month; 30-day readmission was not significantly changed (-0.11%/month, 95% CI -0.73% to 0.50%). Outcome domains: efficiency, quality.	Cochrane EPOC (ITS)	Low risk
Zou et al., 2025 [18]	China; coronary heart disease patients	Interrupted time series (ITS)	C-DRG	Coronary heart disease inpatients	Immediate reduction in low-value coronary revascularization of -9.78% (95% CI -11.08% to -8.48%), with a sustained quarterly decrease of -0.59% (95% CI -0.88% to -0.30%). Outcome domains: coding behavior/service appropriateness.	Cochrane EPOC (ITS)	Low risk
Cao et al., 2024 [16]	Beijing, China (53 hospitals)	Difference-in-differences (quasi-experimental)	Chinese DRG-based prepayment	Patients with neurologic disorders across 53 hospitals	Total costs per case decreased 12.6% and length of stay was reduced by 0.96 days, with significant reductions in in-hospital mortality and readmission. A lower proportion of surgical cases and a decline in the Case Mix Index suggest possible patient selection. Outcome domains: efficiency, quality, coding behavior.	ROBINS-I	Moderate

Source notes: Risk of bias was appraised with the ROBINS-I tool for the difference-in-differences and other quasi-experimental (non-randomized) studies and with the Cochrane EPOC suggested risk-of-bias criteria for the interrupted time series studies (see Appendix B). Reported effect estimates are study-specific and reflect heterogeneous DRG models, settings, currencies, years, and outcome definitions; they should be interpreted in context and not generalized as universal effects. 

Sources cited as background/context (not counted as included evidence): systematic reviews and meta-analyses of DRG-based payment -- for example, Meng et al. [5], Chen et al. [19], Palmer et al. [20], and Pan et al. [21] -- are cited in the Introduction and Discussion to contextualize the primary findings but were not included in the formal evidence synthesis, which was restricted to primary comparative studies. Foundational DRG references (Fetter et al. [1]; Busse et al. [2]), Saudi Arabian health-reform infrastructure documents (the Vision 2030 Health Sector Transformation Program, AR-DRG adoption, the National Platform for Health and Insurance Exchange Services (NPHIES), and the Saudi Billing System, including the coding-validation study by Reka et al. [9], and the PRISMA 2020, SWiM, and other methodological references are likewise retained as background/context references in the Introduction and Discussion only.
